# Effects of Fiddler Crab Burrows on Sediment Properties in the Mangrove Mudflats of Sungai Sepang, Malaysia

**DOI:** 10.3390/biology5010007

**Published:** 2016-01-19

**Authors:** Mohammad Mokhtari, Mazlan Abd Ghaffar, Gires Usup, Zaidi Che Cob

**Affiliations:** 1 School of Environmental and Natural Resource Sciences, Faculty of Science and Technology, National University of Malaysia, UKM Bangi, Selangor D.E. 43600, Malaysia; mmk00188@hotmail.com (M.M.); magfish05@yahoo.com (M.A.G.); giresusup@gmail.com (G.U.); 2 School of Fisheries and Aquaculture Sciences, Universiti Malaysia Terengganu, Kuala Terengganu, Terengganu 21030, Malaysia; 3 Marine Ecosystem Research Centre, Faculty of Science and Technology, National University of Malaysia, UKM Bangi, Selangor D.E. 43600, Malaysia

**Keywords:** bioturbation, multivariate analysis, sediment oxidation, organic content, Principal Component Analysis

## Abstract

In mangrove ecosystems, litter fall accumulates as refractory organic carbon on the sediment surface and creates anoxic sediment layers. Fiddler crabs, through their burrowing activity, translocate oxygen into the anoxic layers and promote aerobic respiration, iron reduction and nitrification. In this study, the effects of four species of fiddler crabs (*Uca triangularis*, *Uca rosea*, *Uca forcipata* and *Uca paradussumieri*) on organic content, water content, porosity, redox potential and solid phase iron pools of mangrove sediments were investigated. In each crab’s habitat, six cores down to 30 cm depth were taken from burrowed and non-burrowed sampling plots. Redox potential and oxidized iron pools were highest in surface sediment, while porosity, water and organic content were higher in deeper sediment. Reduced iron (Fe (II)) and redox potential were significantly different between burrowed and non-burrowed plots. Crab burrows extend the oxidized surface layer down to 4 cm depth and through the oxidation effect, reduce the organic content of sediments. The effects of burrows varied between the four species based on their shore location. The oxidation effect of burrows enhance the decomposition rate and stimulate iron reduction, which are processes that are expected to play an important role in biogeochemical properties of mangrove sediments.

## 1. Introduction

Mangrove sediments are important sinks for organic carbon [1,2,3,4,5]. Mangrove trees are major primary producers in coastal mangrove ecosystems, while other producers like benthic microalgae are deemed less important [6]. As a result, litter fall represents the major organic carbon input into the mangrove sediment [7]. Fallen mangrove leaves lose their liable organic carbon during the early diagenesis process and accumulate as refractory organic matter on the mudflats [1]. Due to poor nutritional value and high amounts of tannin, lignin and cellulose in mangrove detritus, microbial decomposition is slow, especially under anaerobic conditions [8]. Oxygen diffuses less than 1 mm into the superficial layer in mangrove mud surfaces [9]. Below the oxic zone of mangrove sediment, anaerobic microorganisms mediate much of the oxidation of carbon [10]. Sulfate reduction is a dominant decomposition pathway in unbioturbated mangrove sediments [11]. However, it ceases in the presence of more powerful electron acceptors like Fe (III) in the oxidized layers of burrow walls and therefore iron reduction predominates in bioturbated mangrove sediments [12,13,14].

Fiddler crabs are known to increase water content and permeability and reduce median grain size of mudflats [15]. Burrow construction and maintenance displace sediment particles which results in more oxidized sediment with a lower sulfide content and higher mangrove production [13,16,17,18]. Replacing fresh surface sediment with old refractory organic matter from anoxic layers influences sediment decomposition rate and organic matter (OM) [19,20]. Burrows increase the sediment–water interface area in mudflats, thus enhancing chemical and biological exchange rates between water and sediments [18]. Many studies indicate that fiddler crabs effectively reduce organic matter (OM), chlorophyll and meiofaunal density and diversity [19,21,22,23]. As a result, they reduce reactive organic carbon available for other animals. Arújo *et al.* [14] examined the burrow effects of *Uca maracoani* and *Ucides cordatus* and observed different burrow effects between the two species. Despite the broad effects of fiddler crabs on mangrove ecosystem, no attempt has been made to test the difference between burrowed and non-burrowed mudflats with regards to combined effect of depth, habitat and burrow on sediment properties using multivariate analysis. In addition the magnitude of their effects on the sediment properties have not been determined quantitatively. This study aimed to compare the burrowing effects of different species of fiddler crab in one study area. Therefore sediment properties were measured in burrowed and non-burrowed mudflats to quantify the potential role of each species on the mangrove sediment.

## 2. Materials and Methods

### 2.1. Study Site and Field Sampling

**Figure 1:**
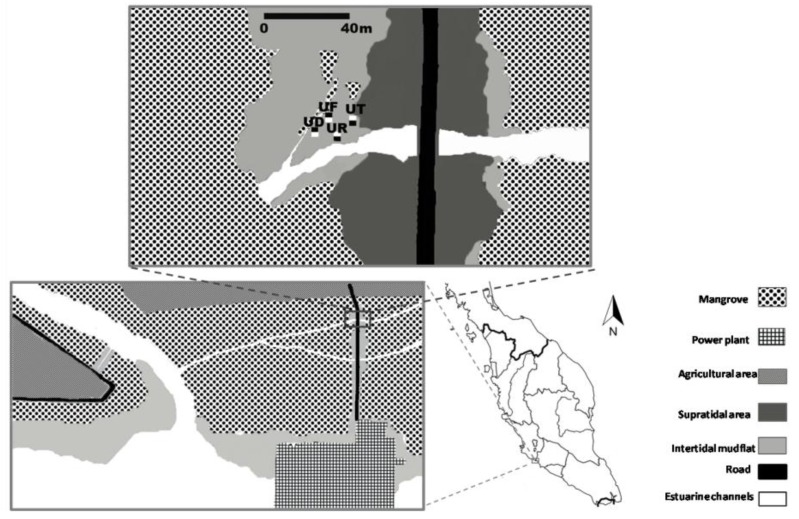
The approximate locations of the *U. paradussumieri* (UD), *U. forcipata* (UF), *U. rosea* (UR) and *U. triangularis* (UT) sampling plots. Burrowed and non-burrowed sediments are indicated by filled (■) and open (□) squares respectively.

This study was conducted in mangrove estuary of the Sungai Sepang, 20 km north of Port Dickson, Peninsular Malaysia. On the mangrove floor, a substrate dominated by a species was regarded as habitat of that predominant species. Habitats of four fiddler crab species including *U. paradussumieri, U. forcipata, U. rosea*, and *U. triangularis* were selected to compare sediment properties between burrowed and non-burrowed areas (Figure 1).

Crab habitats were selected according to the findings of Mokhtari *et al.* 2015 [24]. Accordingly, *U. triangularis* habitat was located near the high tide mark. *U. rosea* habitat was located lower than *U. triangularis* in the intertidal gradient and subsequently *U. forcipata* and *U. paradussumieri* habitats were located on the low shore. *U. paradussumieri* create larger burrows that commonly reach more than 20 cm below the sediment surface, though the burrows of other species of fiddler crabs rarely exceeds 15 cm depth in this area.

At each crab’s habitat, three replicates of 1 m^2^ quadrates were selected as sampling plots for burrowed sediments (B) and another three quadrates (1 m^2^) from non-burrowed sediments (C). Non-burrowed substrates were simply the sediment surfaces within the crab’s habitat, in the vicinity of burrowed sediments where the crabs feed but do not make burrows. The burrow density within burrowed plots was between 25–40 burrows, whereas less than five burrows were commonly observed in non-burrowed plots (1 m^2^). Care was taken to select burrowed and non-burrowed plots with similar conditions including their location along the intertidal gradient. The non-burrowed plots were adjacent to burrowed plots and both plots were located parallel to the tidal water mark and experienced similar tidal inundation and exposure regime. However, due to the proximity of *U. forcipata* habitat to mangrove trees and the presence of pneumatophores in the sampling plots, it was expected that more mangrove litter would be received than other habitats. At each of these quadrats or sampling plots, redox potential was measured separately *in situ* using an Extech RE 300 ORP meter at depth intervals of 0–1, 1–2, 2–3, 3–4, 4–5, 5–6, 8, ….., 226 cm. Correspondingly, three replicates of sediment cores (5 cm diameter) were collected by PVC tubes from each sampling plot. Sediment cores were sealed by rubber stoppers, kept in an ice box and brought to the laboratory for further analyses.

### 2.2. Laboratory Analyses

In the laboratory, sediment cores were removed from the PVC tubes and sliced according to the above mentioned depth intervals, and subsamples were taken from each slice to determine sediment characteristics such as density, porosity, water content, OM, reduced iron (Fe (II)) and oxidized iron (Fe (III)). Sediment density was determined by weighing a known volume of sediment and described as gram per milliliter, water content was measured by weight loss after drying at 70 °C for 12 h, sediment porosity was calculated by the formula (density × water content)/100, and OM was estimated by the loss on ignition method (at 520 °C for 4 h [25]).

In this study, solid phase iron pools, Fe (II) and Fe (III) were a measure of sediment oxidation status, which were determined using the modified method of Lovley and Phillips [26], as described by Kristensen and Alongi [13]. Briefly, 300 mg of sediment was extracted in 5 mL of 0.5 M HCl for 30 min in the shaking platform. After centrifugation (at 3000 rpm for 5 min), 40 µL of aliquots was transferred to cuvets containing Ferrozine to determine the Fe (II). One milliliter of supernatant was transferred to a test tube containing reducing agent hydroxylamine hydrochloride, and was shaken for 15 min before mixing with Ferrozine to determine total reducible iron pools. Therefore, the Fe (III) amount was simply calculated by subtraction of total amount of reducible Fe with Fe (II).

### 2.3. Statistical Analyses

One-way ANOVA followed by a Tukey test were performed to compare crab habitats in terms of sediment density, redox potential, porosity, water and OM. The sediment parameter values were tested for homogeneity of variances using the Bartlett test and accordingly sediment density and OM data were log transformed, due to significant differences in variance among treatments (*p* < 0.01). Principal Component Analysis (PCA) was used to detect the variation of environmental parameters among sediment subsamples. Unlike bivariate analyses (e.g., ANOVA) which are limited to providing a correlation between a pair of variables, PCA has the capability to distinguish between the influences of several variables on the group of samples. PCA transforms the original variables of raw data into the uncorrelated axes known as the principal components. Thus PCA reduces the dimensionality of data and eliminates the redundant information which originates from correlation between sediment parameters. As a result, the reduced space of the first few principal component accounts for most of the data variation. The use of BiPlot in PCA was first introduced by Gabriel [27] which displays observation and variables in one plot. In this study the BiPlots were used to illustrate the correlation between sediment samples and environmental parameters. In BiPlots if the variables line is close to a sample point it will have high impact on it, whereas unimportant environmental parameters are perpendicular to the sample point. The data were mean centered and standardized prior to running the PCA. All statistical analyses were performed by Minitab 16 (Minitab Inc., State College, PA, USA). BiPlots were created by BiPlot add-on for Excel [28].

## 3. Results

**Figure 2:**
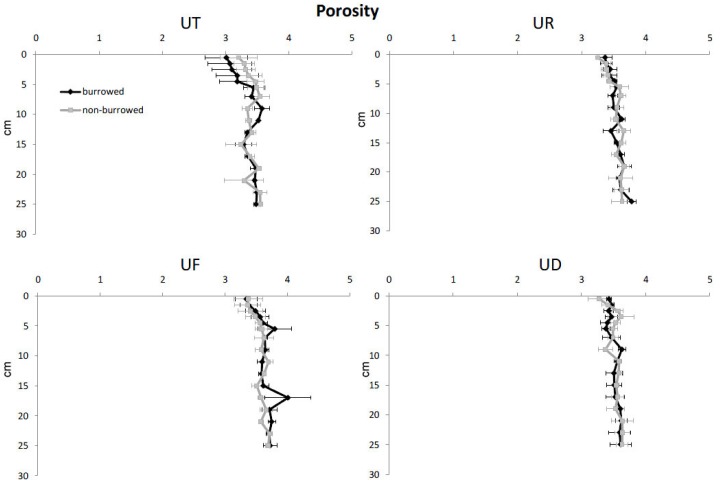
Vertical distribution of porosity in burrowed and non-burrowed sediments of *U. triangularis* (UT), *U. rosea* (UR), *U. forcipata* (UF) and *U. paradussumieri* (UD) habitat.

**Figure 3:**
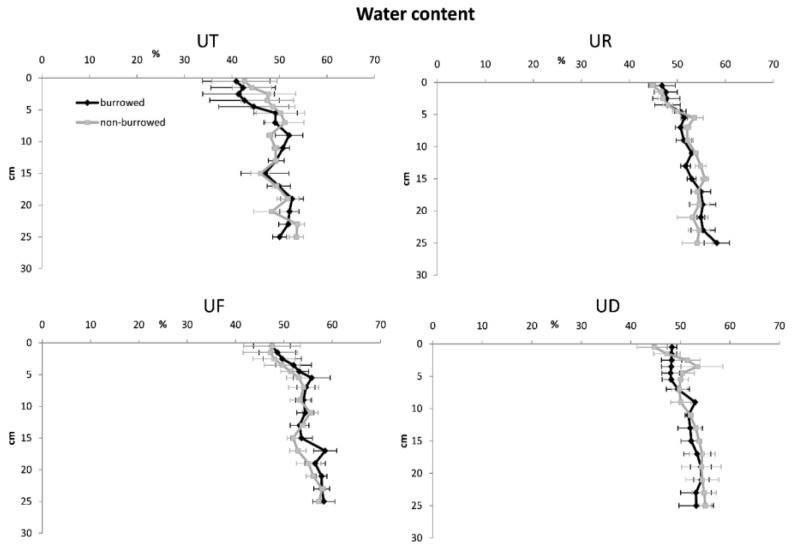
Vertical distribution of water content in burrowed and non-burrowed sediments of *U. triangularis* (UT), *U. rosea* (UR), *U. forcipata* (UF) and *U. paradussumieri* (UD) habitat.

**Figure 4:**
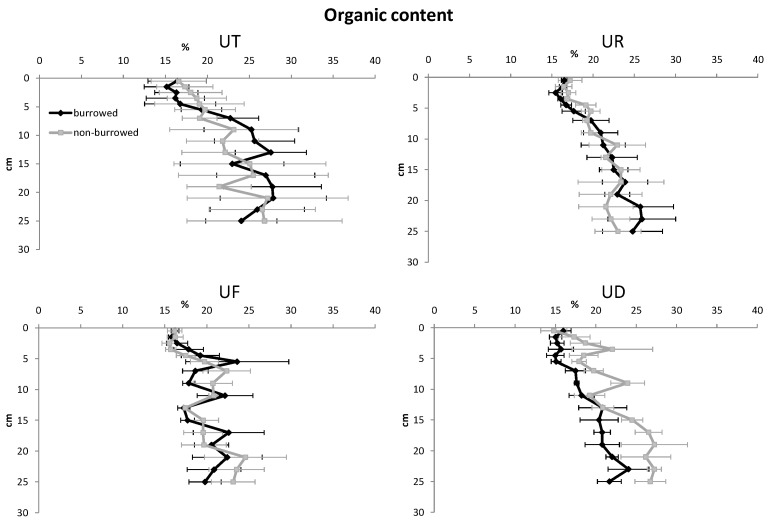
Vertical distribution of organic content in burrowed and non-burrowed sediments of *U. triangularis* (UT), *U. rosea* (UR), *U. forcipata* (UF) and *U. paradussumieri* (UD) habitat.

**Figure 5:**
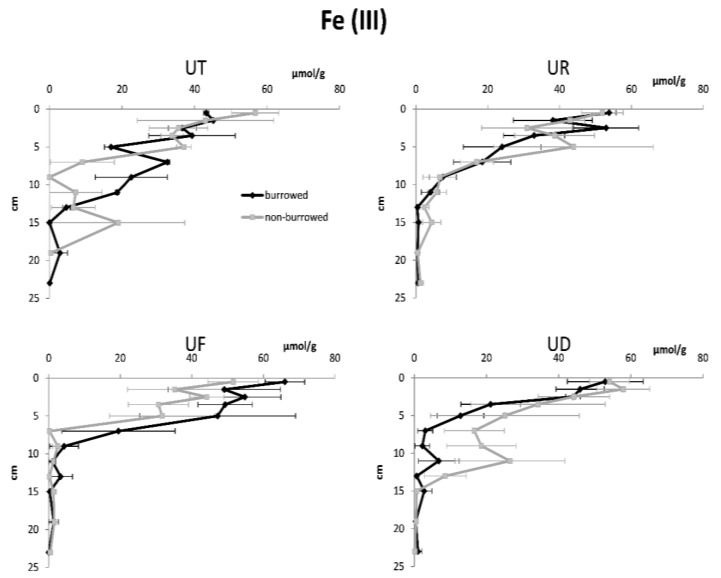
Vertical variation of oxidized iron pools, Fe (III) in burrowed and non-burrowed sediments of *U. triangularis* (UT), *U. rosea* (UR), *U. forcipata* (UF) and *U. paradussumieri* (UD) habitat.

**Figure 6:**
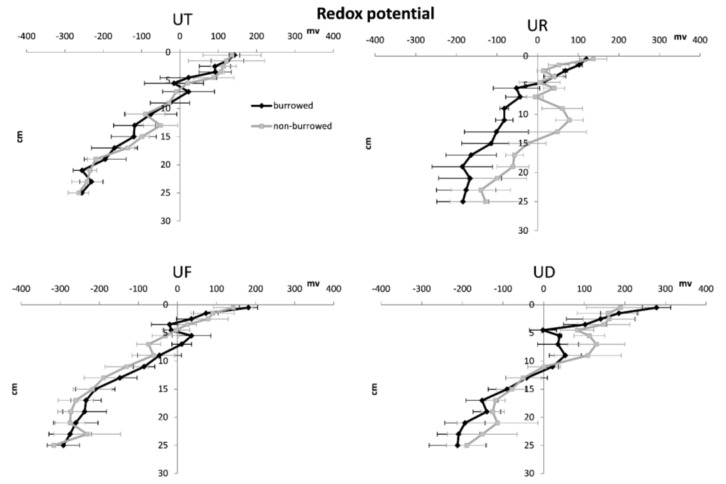
Vertical variation of redox potential in burrowed and non-burrowed sediments of *U. triangularis* (UT), *U. rosea* (UR), *U. forcipata* (UF) and *U. paradussumieri* (UD) habitat.

In almost all sampling plots, sediment porosity, water content and OM increased with increasing depth, while redox potential, sediment density and oxidized iron were higher in surface sediments (Figure 2, Figure 3, Figure 4, Figure 5 and Figure 6). In surface and subsurface sediments down to 4 cm depth, OM and water content varied remarkably between burrowed and no-burrowed plot. Water content was slightly higher in the burrowed plot while lower OM was measured in the burrowed plot (Figure 3 and Figure 4).

No significant differences were detected by ANOVA between burrowed and non-burrowed plots for water and OM, once all sampled depths were considered (Table 1). Analysis of variance indicated that all sediment properties varied significantly with depth (*p* < 0.01). All environmental variables except the iron pools Fe (II) and Fe (III) differed significantly among habitats (*p* < 0.01). However redox potential and reduced iron (Fe (II)) were significantly different between burrowed and non-burrowed mudflats (*p* < 0.01).

**Table 1:** Analysis of variance (ANOVA) for sediment properties among depth, habitat and burrow.

Source of Variation	d.f.	Sum of Squares (SS)	Mean Square (MS)	*F*-Value	*p*-Value
Redox potential					
Depth	15	5,258,508	350,567	49.56	<0.001
Habitat	3	492,236	164,079	23.20	<0.001
Burrow	1	51,557	51,557	7.29	0.007
Error	348	2,461,400	7073		
Total	367	8,267,538			
Sediment density					
Depth	15	18.6837	1.2456	11.22	<0.001
Habitat	3	3.6740	1.2247	11.03	<0.001
Burrow	1	0.0568	0.0568	0.51	0.475
Error	348	38.6340	0.1110		
Total	367	61.1392			
Fe (III)					
Depth	11	91,702.6	8329.5	45.50	<0.001
Habitat	3	144.3	48.2	0.26	0.852
Burrow	1	4.6	4.6	0.03	0.874
Error	245	44,851.1	183.1		
Total	260	136,702.6			
Fe (II)					
Depth	11	6624.48	601.34	11.39	<0.001
Habitat	3	271.37	88.77	1.68	0.172
Burrow	1	502.46	502.46	9.52	0.002
Error	245	12,937.35	52.81		
Total	260	20,335.67			
Porosity					
Depth	15	3.46537	0.23102	5.61	<0.001
Habitat	3	2.53254	0.84139	20.42	<0.001
Burrow	1	0.00073	0.00073	0.02	0.894
Error	348	14.33820	0.04120		
Total	367	20.33684			
Water content					
Depth	15	3030.04	202	10.69	<0.001
Habitat	3	1219.92	403.32	21.34	<0.001
Burrow	1	0.38	0.38	0.02	0.887
Error	348	6578.14	18.90		
Total	367	10,828.49			
Organic content					
Depth	15	3082.75	205.52	9.52	<0.001
Habitat	3	320.66	113.22	5.25	0.001
Burrow	1	58.21	58.21	2.70	0.101
Error	348	7510.79	21.58		
Total	367	10,972.41			

In almost all treatments, Ferric iron (Fe III) was the highest (40–70 μmol/g) at the surface sediment and decreased with depth in accordance with a decrease in redox potential (Figure 5 and Figure 6). Nevertheless, ferrous iron (Fe II) concentration was highest at the intermediate layers, which subsequently decreased with increasing depth and reaches its surface concentration in deeper layers. Most of the iron pools in surface sediment were in oxidized form due to the availability of oxygen. Fe (III) concentration decreased in deeper sediment since it is transformed to reduced form (Fe (II)) in anoxic conditions. In all cases, the amount of Fe (III) showed a higher variability than Fe (II), especially in the top 5 cm sediment layer. Total amount of iron pools decreased with depth in both the burrowed and non-burrowed plots. The concentrations of ferric iron varied considerably between burrowed and non-burrowed plots in *U. forcipata* habitat (Figure 5), indicating the higher oxidation effects of *U. forcipata* burrows.

**Figure 7:**
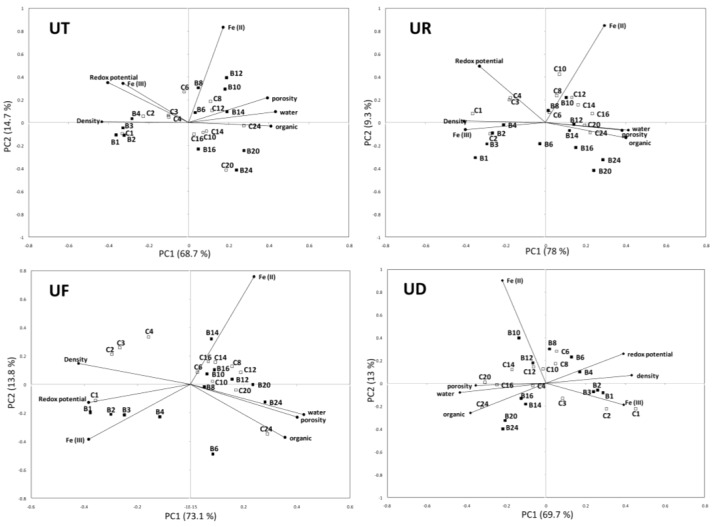
PCA biplot graph of sediment samples from burrowed and non-burrowed mudflats of *U. triangularis* (UT), *U. rosea* (UR), *U. forcipata* (UF) and *U. paradussumieri* (UD) habitat. Sediment samples from the burrowed plot indicated by the letter “B” and closed squares (■). Open squares (□) represent non-burrowed sediment samples (indicated with a “C” in graphs) and numbers refers to the depth layer (cm) of sediment samples.

PCA BiPlots demonstrated that oxidized iron (Fe (III)), redox potential and sediment density were positively correlated with each other and negatively correlated with porosity, OM and water content (Figure 7). Oxidized iron (Fe (III)), sediment density and redox potential decreased with increasing depth, while sediment porosity, water and OM were higher in deeper sediment layers. As a result, the highest vaules of Fe (III), sediment density and redox potentials were observed in surface sediments. In almost all four crab habitats, the top 1 cm surface sample of non-burrowed plots (C) was more similar to subsurface samples down to 4 cm depth of burrowed plot (B) than subsurface sediments of non-burrowed plots.

In *U. paradussumieri* (UD) habitat the surface samples of both burrowed and non-burrowed plots (B1, B2, B3, C1, C2 and C3) were located near the Fe (III) vector (figure 7 UD), indicating that Fe (III) was consistently higher in these samples. Higher variability was found between non-burrowed samples of C1, C2 and C3 compared to burrowed surface samples (B1, B2 and B3). Subsurface layer of non-burrowed plot (C4) was more affiliated with sediment porosity, water and OM than sediment samples from 4 cm depth of burrowed plot (B4). In subsurface layers of between 10 and 12 cm, sediment samples of both burrowed and non-burrowed plots were more associated with Fe (II)vector. In the deepest layers of the burrowed plot, sediment samples of B14, B16, B20 and B24 were affiliated with the OM vector, while non-burrowed samples of C20 and C16 were more affiliated with porosity.

In *U. forcipata* habitat (UF), the sediment samples of 0–1, 1–2, 2–3 and 3–4 cm depth (B1, B2, B3 and B4) from burrowed plots and a surface layer of non-burrowed plot (C1) were more associated with redox potential and Fe (III) vectors. Conversely C2, C3 and C4 samples from the non-burrowed plots were associated with sediment density. At the 6 cm depth, sediment samples of the non-burrowed plots (C6) contained higher amounts of reduced iron than those of the burrowed plots (B6) so that they were more affiliated with the Fe (II) arrow in the PCA plot (Figure 7 UF). In contrast, samples from deeper layers of burrowed plots (e.g., B14) were more affiliated with Fe (II) than non-burrowed samples of same depth. In burrowed plots, the depth of maximum reduced iron was located deeper than in non-burrowed sediments. Sediment samples of B8 from *U. forcipata* habitat (UF) were more associated with porosity and water content than the non-burrowed C8 sample. The deepest samples of the burrowed plots (e.g., B24) were consistent with water and porosity vectors, while non-burrowed samples (e.g., C24) contained higher amounts of OM.

In *U. rosea* habitat (UR), surface layers and subsurface layers down to a 4 cm depth in the burrowed plots, including B1, B2, B3 and B4 together with the top 2 cm layer of the non-burrowed sediments (C1 and C2), were affiliated with the Fe (III) vector. At the 2 cm depth, a high similarity was observed between burrowed and non-burrowed sediments. A thin surficial layer of 1 cm depth from non-burrowed mudflats (C1) was highly associated with the sediment vector. However, redox potential was a consistent variable in sediment samples of 3 and 4 cm layer of non-burrowed sediments (C3 and C4). Intermediate layers of sediment in non-burrowed sediments (C6, C8, C10 and C12) were more affiliated with the Fe (II) vector, while in burrowed plots a thinner layer of intermediate sediments (C8 and C10) was affiliated with reduced iron pools (Fe (II)). The deepest samples of non-burrowed plots were more consistent with sediment porosity, water and OM vectors; however, the deepest samples of burrowed plots (B16, B20 and B24) were negatively correlated with redox potentials.

In *U. triangularis* habitat (UT), surface sediments and subsurface sediments down to 4 cm depth in burrowed plots were highly affiliated with sediment density vector, while in non-burrowed plots only the top 2 cm layer (including C1 and C2) was affiliated with sediment density. Fe (II) was consistently higher in the intermediate layers between 6 and 12 cm of both burrowed and non-burrowed sediments. The deep sediment layers of both burrowed and non-burrowed plots of *U. triangularis* were consistent with OM vector.

The oxidation effect of burrows was limited to the top 4 cm layers of sediment. As in all fiddler crab habitats, the subsurface sediments of burrowed plot, similar to the surface samples, were highly affiliated with density, redox potential and Fe (III) vectors. In addition, the intermediate layer of maximum Fe (II) was narrower and located deeper in the burrowed plot. Consequently, the burrow extends the oxidized surface layer down to 4 cm depth and restricts the suboxic sediment layer where the Fe (II) is maximum.

## 4. Discussion

A lower amount of OM was measured at the oxidized layers of burrowed plot. The decomposition rate of OM is higher under oxic conditions [29]. Accordingly, crab burrows decrease the OM through the oxidation of sediments [30]. In *U. forcipata* habitat, perhaps due to continuous deposition of litter fall under the mangrove trees, the OM did not differ between burrowed and non-burrowed plots. Water content was higher in the top sediment layers of burrowed plot except for *U. triangularis* habitat at the high shore. Rhodes [31] claimed that burrowing activity by reducing sediment hardness increases the water content of sediment. Lower water content in the burrowed plots of *U. triangularis* could be attributed to the location of their burrows in the high shore habitat where the water contents were reduced due to the high evaporation rate at the burrow walls.

Upward diffusion of reduced iron (Fe (II)) and subsequent oxidation resulted in increased Fe (III) in the surface layers [32,33,34]. In the deeper layers, due to the absence of free oxygen and limited amounts of oxidized substances, less iron could be oxidized. Fe (III) sharply decreased down to the 10 cm depth where the redox potential reached negative values (Figure 5 and Figure 6). Decreased Fe (II) under the 14 cm depth is likely caused by the immobilization of Fe (II) with sulfide to form FeS [35]. Below 10 cm depth, redox potential became negative, which is indicative of an oxidized layer where the iron reduction was assumed to reach its maximum value down to a 14 cm depth. In the deeper layers, iron resources became limited and iron reduction became less significant in the total anaerobic respiration.

The results of this study revealed that reduced iron (Fe (II)) was significantly different between the burrowed and non-burrowed plots (Table 1). In *U. rosea* (UR) and *U. forcipata* (UF) habitats, burrowed sediments were more oxidized thussurface and subsurface samples showed a close correlation with redox potential and Fe (III) vectors (Figure 7). Kostka *et al.* [36] similarly found a higher concentration of oxidized iron (Fe (III)) and consequently a higher rate of iron reduction around the *Uca pugnax* burrows. The high rate of iron reduction in mangrove forest is dependent on the oxidation ability of mangrove roots and bioturbation activity of the benthic macrofauna [13]. Kristensen *et al.* [37] estimated that iron reduction involved 80% of carbon oxidation while the sulfate reduction and aerobic respiration accounted for 20% and <6% respectively of total carbon oxidation in bioturbated mangrove sediments of Thailand. Iron reduction is mainly controlled by total iron pools, dissolved organic carbon and availability of oxidized iron, which is mainly fueled by Fe (II) re-oxidation rather than organic carbon accumulation [38]. Therefore, burrowing activity of fiddler crabs which facilitates penetration of oxygen into the sediment layer to re-oxidize Fe (II) is expected to have a great impact on the iron reduction and biogeochemical properties of sediments.

The high values of oxidized iron around *U. forcipata* burrows (Figure 5) could be attributed to the oxidation effects of mangrove roots as well, since *U. forcipata* lives in close proximity to mangrove trees [13,39]. Both *U. forcipata* and *U. paradussumieri* occupy the low intertidal zone near the water channels but *U. forcipata* prefers to live near the mangrove roots [24].

In the habitats of *U. paradussumieri* and *U. triangularis,* the concentration of oxidized iron in the deeper layers (around 10 cm depth) was higher in the non-burrowed sediments (Figure 5). This is comparable with the result of Fe (III) concentration pattern in Thailand’s mangrove forest, where the higher amount of oxidized iron was recorded in the anoxic layers of control treatments [12]. Other burrowing infauna might be responsible for the higher oxidized iron pools recorded in the non-burrowed sites. The activity of fiddler crabs, either by grazing or disturbance, might decrease the abundance of other burrowing infauna [19,40,41].

## 5. Conclusions

The results of this study revealed that redox potential and reduced iron pools were significantly different between burrowed and non-burrowed sediments. The subsurface layers of burrowed sediments were significantly oxidized by the crabs’ burrowing activity. Crab burrows reduced the organic matter of the sediments particularly at the top 4 cm layer where the oxidation effect of burrowing is most evident. Except for *U. triangularis*, crab burrows increase water content of the sediment. The results of this study indicated that the effects of burrows on sediment properties depend on the location of the burrows on the shore, as the tidal inundation and exposure time changes along the intertidal gradient. The current study focused on the effects of fiddler crab burrows on sediment properties at the relatively coarse scale of 1 m^2^ sampling plots. For a better understanding of burrow effects, a more detailed study with a finer sampling design at the scale of individual burrow walls is required.
